# Differential pattern of deposition of nanoparticles in the airways of exposed workers

**DOI:** 10.1007/s11051-016-3711-8

**Published:** 2017-01-17

**Authors:** Elizabeth Fireman, Rinat Edelheit, Moshe Stark, Amir Bar Shai

**Affiliations:** 1 Laboratory of Pulmonary and Allergic Diseases, Tel Aviv University, Tel Aviv, Israel; 2 Pulmonology Department, Tel-Aviv Sourasky Medical Center affiliated to the Sackler Faculty of Medicine, Tel Aviv University, 6 Weizman Street, 64239 Tel Aviv, Israel; 3 Department of Occupational and Environmental Health School of Public Health, Sackler Faculty of Medicine, Tel Aviv University, Tel Aviv, Israel

**Keywords:** EBC, Induced sputum, Ultrafine particles, Nanoparticle exposure, Aerosols, Environmental and health effects

## Abstract

Ultrafine particles (UFP) have been postulated to significantly contribute to the adverse health effects associated with exposure to particulate matter (PM). Due to their extremely small size (aerodynamic diameter <100 nm), UFP are able to deposit deep within the lung after inhalation and evade many mechanisms responsible for the clearance of larger particles. There is a lack of biologically relevant personal exposure metrics for exposure to occupational- and environmental-related micro- and nano-sized PM. The aim of the present study is to assess UFP in induced sputum (IS) and exhaled breath condensate (EBC) as possible biomarkers for assessing lung function impairment. Sputum induction and EBC testing were performed by conventional methods. UFP particles were assessed with the NanoSight LM20 (NanoSight Ltd, London, UK). The subjects included 35 exposed and 25 non-exposed workers. There were no group differences in pulmonary function test results and differential cell counts, but 63.6% of the exposed subjects had a higher percentage of neutrophils (OR3.28 *p* = 0.03) compared to the non-exposed subjects. The exposed subjects had higher percentages of UFP between 10 and 50 nm (69.45 ± 18.70 vs 60.11 ± 17.52 for the non-exposed group, *p* = 0.004). No differences were found in the IS samples. Years of exposure correlated positively to UFP content (*r* = 0.342 *p* = 0.01) and macrophage content (*r* = −0.327 *p* = 0.03). The percentage of small fraction of UFP in EBC, but not IS, is higher in exposed workers, and EBC may be a sensitive biomarker to assess exposure to nanoparticles.

## Introduction

Exposure to dust in the workplace is associated with a variety of pulmonary and systemic illnesses. The reactions that occur within the lungs vary with the size of the dust particle and its biologic activity. Anthropogenic sources, primarily involving the combustion of fossil fuels, account for a significant proportion of nanometer–diameter aerosols in urban areas, and many industrial processes, including welding, smelting, and the use of diesel engines, lead to the production of airborne particles in the nanometer-size range (Harrison et al. 2000). Particulate material (PM) in ambient air pollution is characterized as being coarse (PM_10_, aerodynamic diameter range 2.5–10 μm), fine (PM_2.5_, 2.5–0.1 μm), and ultrafine (UFP, nano-sized, <0.1 μm). In occupational hygiene, however, it is common to differentiate manufactured nanoparticles from ultrafine particles (UFP) derived from natural, human, or industrial sources (Debia et al. 2013). Epidemiology has identified correlations between exposure to UFP and increased pulmonary and cardiovascular morbidity and mortality (Strak et al. 2010; Sioutas et al. 2005; Meier et al. 2015). It has been postulated that upon inhalation, UFP and nanoparticles are increasingly toxic compared to larger particles because of their increased reactivity, surface area, particle number mass, deposition potential, and ability to translocate to other organ systems, such as the cardiovascular and/or neuronal system, and elicit adverse effects (Frampton 2001).

Most of the above-cited studies were based on environmental monitoring data, animal models, and controlled human exposures. There is a lack of biologically relevant personal exposure metrics for exposure to occupational- and environmental-related particulate matter. We demonstrated that analysis of particles recovered by induced sputum (IS) can serve as a biological monitoring method in addition to the traditional occupational parameters in several studies (Fireman et al. 1999; Fireman et al. 2004; Stark et al. 2014; Fireman et al. 2014; Fireman et al. 2008). Those studies were performed solely on IS samples and by measuring particle size distribution in the micro range scale (0.5–60 μm). We present here, for what we believe to be the first time, an analysis of UFP particles measured in both IS and exhaled breath condensate (EBC) in order to sample UFP from two anatomical compartments. The UFP that were measured in EBC represent the epithelial lining fluid, and the UFP from IS represents the inner epithelial compartment) (Geiser and Kreyling 2010).

Only few studies have looked at “endogenous” particles in the EBC of smokers (Bredberg et al. 2013) and occupational expose workers (Olin 2012) as a biomarker of inflammatory changes in airway disease that should reflect alterations in the respiratory tract lining fluid, whose main constituents are lipids and proteins. The particles measured in those studies, however, are only in the micro range size (coarse PM_10_ particles). To date, there have been no reports on individual measurements of UFP content in workers exposed to hazardous particulate matter. The purpose of the current study was to examine the dynamic differences between UFP content in IS vs UFP content in EBC and to determine whether exhaled UFP are correlated with symptomatic, functional, and/or laboratory measures and can serve as useful biomarkers for early detection of impairments in lung function in exposed workers.

## Materials and methods

### Study population

The study included 60 participants who were divided into two groups according to the presence of exposure. The exposed group included 25 industrial workers who were exposed to occupational dust from industrial sources while executing their work. The non-exposed control group included employees who were not exposed to any kind of dust during their work. Exposure and demographic data were retrieved by a questionnaire followed by a personal interview. The participants were adults over the age of 20 years who came to the Laboratory of Pulmonary and Allergic Diseases, Tel-Aviv Sourasky Medical Center, for a medical assessment related to various respiratory symptoms. They underwent sputum induction, EBC testing, and pulmonary function testing.

### Pulmonary function tests

Pulmonary function tests (PFTs) were performed by a Masterlab spirometer (Masterlab, E. Jaeger, Wurzburg, Germany). Measurements were carried out according to standard protocols of the American Thoracic Society (ATS) guidelines (Miller et al. 2005), and the results were expressed as percent predictive value. The best of three consecutive measurements was chosen. Forced expiratory volume in 1 s (FEV1) and forced vital capacity (FVC) were recorded. The FEV1 result and the FEV1/FVC ratio were considered low if they were less than 80% of expected or less than a ratio of 0.75, respectively.

### IS collection and processing

IS was obtained as previously described in detail elsewhere (Fireman et al. 1999). After pretreatment with a short-acting β-2 agonist, 3% saline was administered by a nebulizer (U1 Ultrasonic Nebulizer; Omron HealthCare, Henfield, West Sussex, UK) for up to 20 min while the subjects were encouraged to cough and expectorate sputum into a sterile container. Samples were stored at 4 °C and processed within 3 h. All portions with little or no squamous epithelial cells (i.e., a rich non-squamous epithelial cell fraction considered to originate from the lower respiratory tract, hereafter referred to as “plugs”) were collected and treated with dithiothreitol [DTT (Sputolysin); Calbiochem Corp, San Diego, CA, USA]. The cell suspension was filtered through a 52-μm nylon gauze, and the effect of DTT was stopped by diluting the suspension with phosphate-buffered solution to a volume equal to the sputum plus DTT. After centrifugation, the supernatants were resuspended and cytospinned (Shandon Southern Instruments, Sewickley, PA, USA), and the slides were stained with Giemsa.

Sputum was considered eosinophilic if at least 2.7% of the sputum cells were eosinophils and neutrophilic if at least 65% of cells were neutrophils.

### EBC collection

EBC was collected by a TurboDECCS device (Medivac, Parma, Italy) according to the manufacturer’s instructions (Goldoni et al. 2013). Briefly, the subjects were asked to breathe into the collecting system for 5 min under conditions of normal tidal volume and without a noseclip. All of the samples were stored at −80 °C until analysis. All EBC collections were performed in a controlled temperature (22–23 °C) and humidity (50%) by a closed air conditioning system.

### Ultrafine particle measurement

The particle size number distribution of PM_0.1_ was assessed in the EBC samples with the NanoSight LM20 system (NanoSight Ltd., Salisbury) using the Nanoparticle Tracking Analysis (NTA) method of visualizing and analyzing particles in liquids that relates the rate of Brownian motion to particle size. The rate of movement is related only to the viscosity of the liquid, the temperature, and the size of the particle and is not influenced by particle density or refractive index. The particles contained in the sample can be visualized by virtue of the light they scatter when illuminated by a laser light source. The technique retrieves samples from a volume of 120 × 80 × 20 microns. Sample concentration is adjusted (10^7^–10^9^ particles/ml) such that a significant number of particles is present in the beam path. Approximately 0.3 ml of EBC was introduced into the viewing unit using a disposable syringe. Three videos of 30-s duration were recorded and analyzed (NTA software version 2.0) for each sample. Total particle count, percent of particles that were in the nano-sized range, and total nano-sized particle counts were recorded.

### Statistical analysis

Continuous PFT data, IS deferential cell counts, exposure duration, and age were correlated to exposure by the *t* test, while categorical data and exposure were assessed by the *χ* ^2^ test. Each measurement of PM_0.1_ in the EBC and in the IS samples were expressed as the average of three consecutive measurements. The frequency of particle ranges (10–50 nm) were combined to one parameter for EBC and IS and associated to exposure status by the *t* test. A dichotomy cutoff (61%) was used in a logistic regression model to evaluate an association to 10–50 nm particle exposure status. The within-subject effect of particles 10–50 nm in sputum and in EBC was correlated to exposure by the ANOVA test repeated measures. GLM repeated measurements were done using mixed models, and the exposure was used for the between-subject factors and the particles for the within-subject factors. The measurements were done twice in the sputum and EBC samples, respectively.

## Results

The demographic and particle characteristics of the studied population are shown in Table 1. The exposed subjects were older than the non-exposed subjects (55.64 ± 14.19 vs 48.08 ± 14.49 years, respectively), had worked for more years (26.36 ± 15.86 × 14.49 vs 12.53 ± 1.8 years, *p* ≤ 0.01) and were composed mostly of men (20 vs 14 in the non-exposed group *p* ≤ 0.01).

**Table 1:** Demographic and particle parameters of study

	Exposed	Non-exposed	*p* value
Age (years)	55.64 ± 14.19	48.08 ± 14.49	0.04
Work duration	26.36 ± 15.86	12.53 ± 1.8	<0.01^a^
Gender (male)	80 (20)	40 (14)	<0.01^b^
Work duration	26.3615 ± 15.86	12.53 ± 1.8	<0.01^a^
EBC 10–50 (%)	62.17 ± 29.22	35.55 ± 22.90	<0.01^c^
Sputum 10–50 (%)	56.42 ± 29.61	76.61 ± 33.05	<0.01^c^
Total EBC 100 nm^d^	87.67 ± 41.73	58.75 ± 35.166	<0.01 ^c^
Total (IS) 100 nm^d^	69.53 ± 34.85	94.92 ± 38.32	<0.01 ^c^

Exposure data were evaluated by questionnaire interviews

^a^Differences between non-exposed and exposed workers were evaluated by the *t* test

^b^Differences between non-exposed and exposed workers were evaluated by the *χ* ^2^ test

^c^Differences between non-exposed and exposed workers were evaluated by the Mann–Whitney test

^d^Total number of particles under 100 nm

Percent particles between 10 and 50 nm and percent total particles under 100 nm were higher in the EBC samples for exposed workers and vice versa for IS samples (*p* < 0.01).

The types of jobs for all study participants are listed in Table 2.

**Table 2:** Occupational background of the study population

Type of job among the exposed workers	Type of job among the non-exposed workers
Seamstress (*n* = 1)	Salesperson (*n* = 1)
Baker (*n* = 1)	Office manager (*n* = 3)
Cook (exposure to smoke) (*n* = 1)	Adviser (*n* = 1)
Metal worker (*n* = 1)	Psychologist (*n* = 1)
Dental technician (*n* = 1)	Secretary (*n* = 5)
Worker in paper mill (*n* = 1)	Researcher (*n* = 1)
Carpentry (*n* = 1)	Musician (*n* = 1)
Stone mason (exposure to silica (*n* = 7)	Confectioner (*n* = 1)
Auto mechanic (*n* = 1)	Scriptwriter (*n* = 1)
Electrical technician (*n* = 1)	Assistant in kindergarten (*n* = 1)
Welder (*n* = 1)	Waitress (*n* = 1)
Bookbinder (*n* = 1)	Housewife (*n* = 1)
Laundry worker (*n* = 1)	Security guard in school (*n* = 1)
Diamond polisher (*n* = 2)	Designer (*n* = 1)
Gas station attendant (*n* = 1)	Translator (*n* = 1)
Cosmetician (*n* = 1)	Nurse (*n* = 1)
Engineer (*n* = 2)	Social worker (*n* = 1)
	Maintenance worker (*n* = 3)
	Private investigator (*n* = 1)
	Unemployed with no past exposures (*n* = 6)

**Fig. 1:**
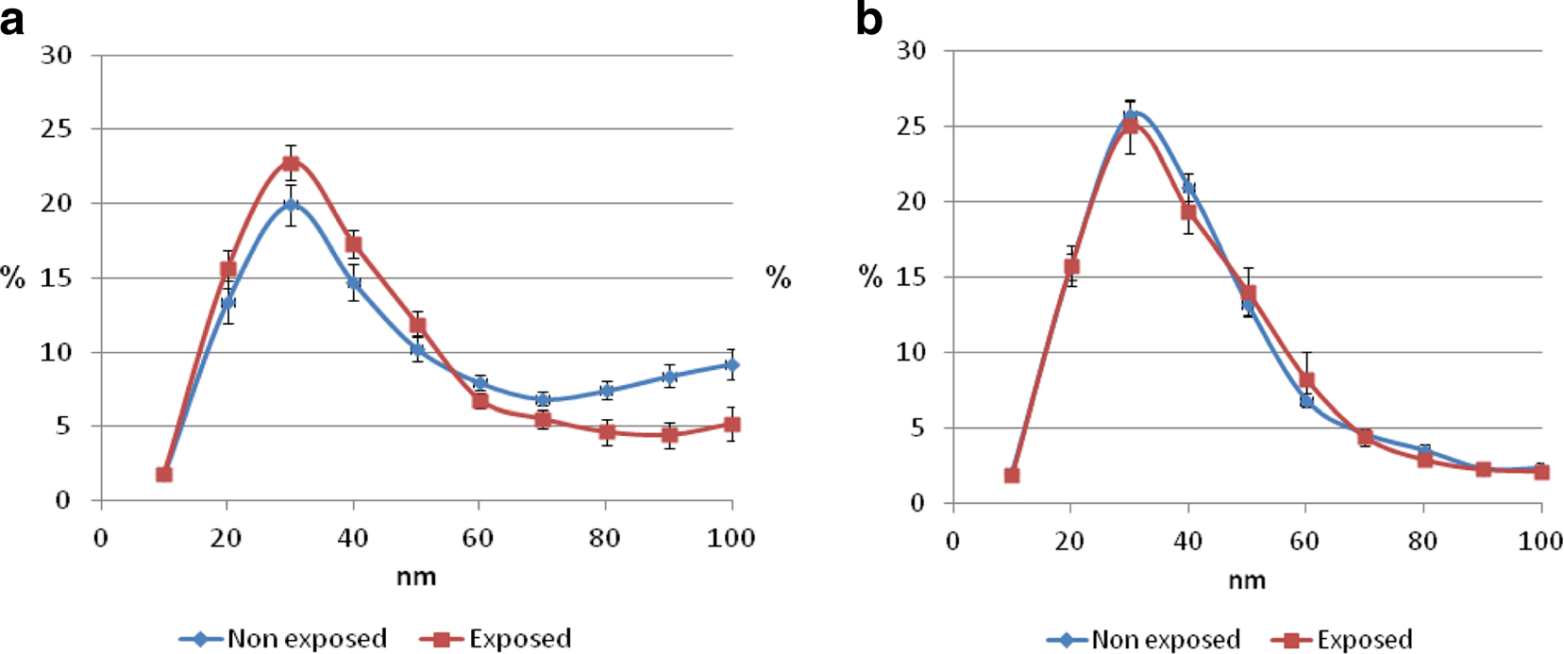
**a** Frequency of UFP in EBC. **b** Frequency of UFP in IS. UFP were evaluated by NanoSight LM20 in the sputum specimens. The frequency of EBC UFP (**a**) and IS UFP (**b**) for each size (nm). The *x*-axis represents the particle size (nm), and the *y*-axis represents the frequency for each size

The PFT results were not significantly different between the two groups (Table 3). The differential cell counts in the exposed group had a higher percent of neutrophils (63.03 ± 24.91 vs 52.66 ± 22.83 for the non-exposed group, *p* = NS) and lower percentage of macrophages 16.21 ± 15.88 vs 27.16 ± 23.04, respectively, *p* = 0.04). The frequency of UFP in EBC and IS are shown in Fig. 1a, b. The frequency of UFP in IS was almost identical for both groups. In contrast, a different pattern was observed in the EBC results: they were higher in the fraction of 10–50 nm among the exposed subjects compared to the non-exposed ones (69.45 ± 18.70 vs 60.11 ± 17.52, respectively, *p* = 0.004).

**Table 3:** Pulmonary function testing and differential cell counts of the exposed and non-exposed workers

	FVC (%)	FEV1 (%)	FEV1/FVC	
Exposed (*n* = 25)	87.29 ± 18.50	85.15 ± 17.84	77.86 ± 8.86	
Not exposed (*n* = 34)	89.29 ± 16.48	84.59 ± 21.56	75.89 ± 12.99	
*p* value	0.65	0.41	0.29	
	Neutrophils %	Lymphocytes %	Macrophages %	Eosinophils %
Exposed (*n* = 25)	63.03 ± 24.91	10.28 ± 5.06	16.21 ± 15.88	6.23 ± 13.0.9
Non-exposed (*n* = 34)	52.66 ± 22.83	12.15 ± 7.65	27.16 ± 23.04	8.12 ± 13.10
*p* value	0.11	0.27	0.04	0.09

Exposure was evaluated by questionnaire interviews. Pulmonary function test results were evaluated by conventional methods. PFTs parameters are represented as percentages of the predicted value. Differences between non-exposed to exposed subjects were evaluated by the *t* test. Differential sputum cell counts were done on 400 non-squamous cells stained by Giemsa staining. Differences between non-exposed to exposed subjects were evaluated by the *t* test

*FEV1* forced expiratory volume in 1 s, *FVC* forced vital capacity

Neutrophilic inflammation is an important biomarker in occupational exposures. In this context, we divided individuals according to a cutoff of 61% neutrophils in the differential cell counts in IS. The results showed that 65.2% of the exposed individuals displayed a neutrophilic pattern compared to only 36.4% of the non-exposed ones (OR 3.28 *p* = 0.03) (Table 4). Moreover, an increase in percentage of UFP fraction of 10–50 nm had an OR of 1.08 (*p* = 0.006) (Table 5) to belong to the group of neutrophilic inflammation and vice versa having neutrophilic inflammation predict high risk to accumulate nanoranged particles (fraction of 10–50 nm). The fraction of 10–50 nm in IS did not show a comparable correlation (Fig. 1b). The length of exposure correlated positively to the UFP in EBC (*r* = 0.342 *p* = 0.01) and macrophage percentage (*r* = −0.327 *p* = 0.03) (Tables 1 and 6).

**Table 4:** Differentials OR of inflammatory and functional parameters in exposed vs non-exposed subjects

	Neutrophil		FEV1/FVC	
	<61%, *n* (%)	>61%, *n* (%)	<75%, *n* (%)	>75%, *n* (%)
Exposed (*n* = 25)	8 (34.8)	15 (65.2)	15 (44.1)	19 (55.9)
Non-exposed (*n* = 34)	21 (63.6)	12 (36.4)	11 (44)	14 (56)
*p* value	0.03 OR 3.28 95% CI (1.07–9.98)		0.99 OR 1.00 95% CI (0.35–2.84)	

The differential sputum cell count was done on 400 non-squamous cells stained by Giemsa staining. Neutrophil percentages in sputum were converted to dichotomy parameter with a cutoff of 61%. FEV1/FVC was converted to dichotomy parameters with a cutoff of 75%. Association between non-exposed and exposed subjects to those parameters were evaluated by the *χ* ^2^ test

**Table 5:** Differential OR of EBC, IS, UFP, and inflammatory parameters in the study population

	B	OR	CI	*p* value
EBC 10–50 nanoparticles	0.08	1.08	1.02–1.14	0.006
Sputum 10–50 nanoparticles	−0.01	0.98	0.87–1.10	0.755
Neutrophils >61%	1.64	5.18	1.28–20.91	0.021

The association between nanoparticles in EBC and sputum and percent neutrophils to exposure was done by multivariate logistic regression

**Table 6:** Correlation between length of exposure to ultrafine particles and inflammatory and functional parameters

Parameter	Variable	Spearman *R* correlation	*p* value
Particles of 10–50 nm	Years of exposure	0.342	0.01
FEV1/FVC	Years of exposure	−0.127	0.33
Macrophages %	Years of exposure	−0.327	0.03
EBC 10–50 nm	Forced vital capacity	−0.255	0.05

Data on years of exposure were derived from questionnaires and interviews

The gradient of UFP in IS and EBC showed a marked difference between exposed vs non-exposed individuals, being much higher in the latter (59.8 ± 2.68 vs 79.87 ± 1.12 in IS and 72.34 ± 3.19 vs 79.17 ± 1.34, respectively, *p =* 0.01) (Tables 1 and 7).

**Table 7:** Interaction between UFP in EBC and IS

		Mean	Std. error	95% confidence interval		*p* value
				Lower bound	Upper bound	
Non-exposed subjects	EBC	59.83	2.68	54.45	65.20	<0.01
	Sputum	79.87	1.12	77.60	82.13	
Exposed subjects	EBC	72.34	3.19	65.94	78.74	
	Sputum	79.17	1.34	76.48	81.87	

The interaction between UFP particles (10–50 nm) in EBC and in sputum specimens was evaluated by GLM repeated measurements as described in the “Materials and Methods” section. Values were adjusted to age and gender

## Discussion

There are many concerns that nano-sized materials might introduce health risks upon occupational and consumer exposure. Although many of them are produced, handled, and present in fluids during aerosolization of energetic processes, such as vortexing, weighing, sonication, mixing, and blending, no studies have estimated the internal personal-level burden of UFP by means of biological monitoring. To the best of our knowledge, we now present the first study to measure UFP in biological samples in an occupational setup. In the current investigation, we *directly* measured particulate matter in IS and EBC samples from the airways of exposed individuals compared to non-exposed individuals with the aim of finding a biomarker that can monitor UFP accumulation as a marker of exposure in correlation to functional and inflammatory parameters.

We had previously shown that combined IS and EBC measurements detect underlying inflammation in airways of asymptomatic welders. It emerged that the particle burden (higher % particles >2 mm in diameter), inflammatory cells (higher % neutrophils), and level of oxidative stress (H_2_O_2_ in EBC) were a function of the type and the duration of welding (Fireman et al. 2008). Moreover, we demonstrated that biological monitoring by IS indicated that a >92% accumulation of <5-μm particles correlated significantly to a positive beryllium lymphocyte proliferation test result in a group of 63 Israeli and 37 American workers exposed to hazardous dust containing beryllium (OR 3.8, 95% CI 1.2–11.4, *p* = 0.015) among all participants with a follow-up of 2 years (Fireman et al. 2014).

This is our first report dealing with individual measurements of UFP content in workers exposed to hazardous particulate matter. We tested exposed vs non-exposed individuals referred because of respiratory symptoms. The exposed population was older, had fewer females, and had accumulated more years in the workplace than the non-exposed population. These results are compatible with other publications that showed that patients with a history of high exposure to gases/fumes were less likely to be women and that those with high biological dust exposure were more likely to be older (Rodríguez et al. 2014). The same was shown in exposures to carbon black dust (Neghab et al. 2011).

In our current study, the participants had been exposed to a variety of hazardous particulate matter, and the results revealed that there is a general delay of many years between occupational exposures and the development of pulmonary disease. Our study population represents a unique cohort of workers that were referred for respiratory evaluation after many years of exposure. The initial respiratory symptoms were present, but there was no functional impairment. In fact, there were no differences in pulmonary function parameters between the exposed and the non-exposed individuals.

The findings of the present study emphasize the role of biomarkers involved in inflammatory processes and in the biomonitoring of particulate matter for early detection of injury due to occupational exposure. These findings may be of considerable epidemiological relevance for the assessment of ultrafine particle content.

We had recently evaluated the effect of individual exposure to UFP on functional respiratory parameters and airway inflammation in 52 children aged 6–18 years who had been referred for assessment due to respiratory symptoms. We found that the total and percent of UFP content correlated with wheezing, breath symptom score, and sputum eosinophilia (Benor et al. 2015). The eosinophils we found were not correlated to any of the parameters we studied.

Neutrophils comprise the main type of cells that correlates with occupational exposures, as we previously demonstrated in our study on welders (Fireman et al. 2008) and firefighters (Fireman et al. 2004). The cutoff for abnormality for neutrophils was 61%. This choice was based on a recent unpublished study (2005–2012) on the leukocyte counts of 905 Israeli patients diagnosed as having various pulmonary diseases: We adopted this value at the cutoff value for the current investigation. A very recent study on Indian goldsmiths found that neutrophils in sputum correlated with occupational cadmium exposure, spirometry, and lung cell DNA damage (Moitra et al. 2015). In contrast with our earlier findings and those of others, in the current work, we *directly* demonstrated that this neutrophilic inflammation is correlated with UFP retrieved from EBC samples but not from UFP recovered from IS samples. Moreover, IL-8, the classical cytokine that induced chemotaxis for neutrophils, was significantly higher in supernatants recovered from IS samples in the exposed workers compared to the non-exposed (data not shown). This raised the interesting question about the patterns of deposition of UFP particles in the airways and their internalization pathway. All of our exposed subjects were actively employed, and the IS and EBC sampling was done within 24 h from their last exposure at the workplace. The exposure was chronic and continued over extended periods of time. In the exposed workers, the small fraction of UFP (10–50 nm) were retained in the epithelial lining fluid and recovered by EBC, while the largest fraction of UFP (50–100 nm) were rapidly translocated to inner compartments. It is known that after particle inhalation and their deposition on the lung epithelium, the retention of particles starts with their wetting by surfactant and the epithelial lining fluid and their subsequent displacement from the air into the aqueous phase regardless of particle shape, surface topography, and surface-free energy (Geiser et al. 2003). Our results support the statement by Moller et al. that the probability of long-term particle retention in the airways is inversely correlated to particle size as was shown in both of our study groups. The amount of retained fraction (10–50 nm), however, was higher in our exposed group. This may be due to the different degrees of airway inflammation among the subjects in both groups. Our exposed workers showed here a clear neutrophilic pattern due to exposure. In this context, we recently showed that the pattern of distribution of UFP that were recovered from the bronchoalveolar lavage of mice exposed to smoke was dependent upon the inflammatory status of the animals and that the retention or translocation of UFP through the alveolar barrier was highly dependent upon inflammatory status (Bar-Shai et al. 2015).

The main limitations of the research are the heterogeneity of the study group and a lack of chemical analysis of particles. We did not analyze metal contents in the present study, but the results of our lab’s earlier analysis of metals in EBC samples by X-ray fluorescence showed that they had not differed between exposed and non-exposed workers (supporting the contention that the main effect of the very small particles is caused by the neutrophilic inflammation and not by the type of metal).

Based on the clear differences that we found between the exposed and non-exposed participants of this study, we are confident that our method may offer new opportunities in the new field of nanomedicine for the diagnosis and therapy of pulmonary airway disease caused by exposure to airborne nano-sized particulate matter.

## Conclusions

This study is the first to report a direct dosimetry as well as describe exposure metrics for identifying very small particles recovered from the airways of exposed workers. Nanoparticles may play a crucial role in future surveillance programs in terms of elucidating obscure toxicity resulting from exposure in multiple industries.
